# ‘EngNT’ — Engineering live neural tissue for nerve replacement

**DOI:** 10.1042/ETLS20210085

**Published:** 2021-07-23

**Authors:** James B. Phillips

**Affiliations:** 1 Chief Scientific Officer, Glialign Ltd, UCL Business, 97 Tottenham Court Rd, London W1T 4TP, U.K.; 2 Professor of Regenerative Medicine, UCL Centre for Nerve Engineering, Department of Pharmacology, UCL School of Pharmacy, 29–39 Brunswick Square, London WC1N 1AX, U.K.

**Keywords:** advanced therapy, neuroregeneration, regenerative medicine, tissue engineering

## Abstract

Peripheral nerve injury can result in severe long-term disability and current clinical approaches for repairing large gaps rely on the nerve autograft. Engineered Neural Tissue (EngNT) has been developed to provide living aligned therapeutic cells in a stabilised collagen hydrogel, mimicking the key features of the autograft. This Perspective article will introduce the field and discuss the current stage of translation, highlighting the key opportunities for commercial and clinical development.

## Introduction

Peripheral nerves connect to and control the tissues and organs of the body. Nerve injury can be debilitating, resulting in motor and sensory losses, disruption to autonomic functions and chronic pain. Trauma e.g. due to road traffic collisions or violence, is a leading cause of nerve damage, resulting in physical disability as well as impacting on personal and societal interactions for those affected [1]. Incidence of nerve injury is difficult to determine accurately but it is likely to affect many millions of people globally every year [2–4].

Nerves are effectively bundles of axons that conduct action potentials to and from target organs, with the neuronal cell bodies located within or adjacent to the CNS. Cut nerves form two distinct compartments, an upstream proximal segment containing truncated axons still connected to the CNS, and a downstream distal segment in which the axons degenerate [5]. Surrounding the axons are the Schwann cells, which adopt a repair phenotype following damage. In the distal segment of a damaged nerve the columns of repair Schwann cells encourage neuronal regeneration, providing a supportive chemical and physical environment through which axonal sprouts can extend [6,7]. Ultimately this can result in reconnection to the target tissues, however, physical continuity must be restored at the injury site before regeneration can progress.

Where discontinuity can be repaired surgically through directly reconnecting proximal and distal nerve stumps without undue tension then that approach is favoured. However, many nerve injuries result in gaps which must be bridged [8,9]. The approach used in such cases tends to be the nerve autograft, where a section of nerve tissue is harvested from elsewhere in the patient's body and grafted to bridge between the proximal and distal nerve stumps. The donor tissue provides columns of repair Schwann cells and aligned tissue architecture that can support effective regeneration across the gap, but this approach is associated with donor site morbidity and the amount of suitable nerve tissue available is limited [10,11]. While various biomaterial conduits and decellularized nerve tissue options have been developed as alternatives, they lack the living Schwann cells that are required to support and direct regeneration across long distances [12,13].

This situation presents an attractive opportunity for tissue engineers. Peripheral nerves are unusual in their potential to *regenerate* following trauma, where many other tissues tend to *repair* with scarring following damage [14]. The latter is particularly true in other parts of the nervous system such as the brain and spinal cord where regeneration is limited [15]. Since peripheral neurons regenerate well through freshly denervated nerve tissue (e.g. in the distal stump or an autograft), recapitulating that environment in an engineered tissue should provide an excellent replacement to the current autograft approach [16–18]. As tissue engineering techniques and understanding of peripheral nerve biology have matured over recent decades, and clinical application of Advanced Therapy Medicinal Products (ATMPs) has become more established, the feasibility of constructing living artificial tissue to bridge gaps in peripheral nerve repair has improved.

**Figure 1.**
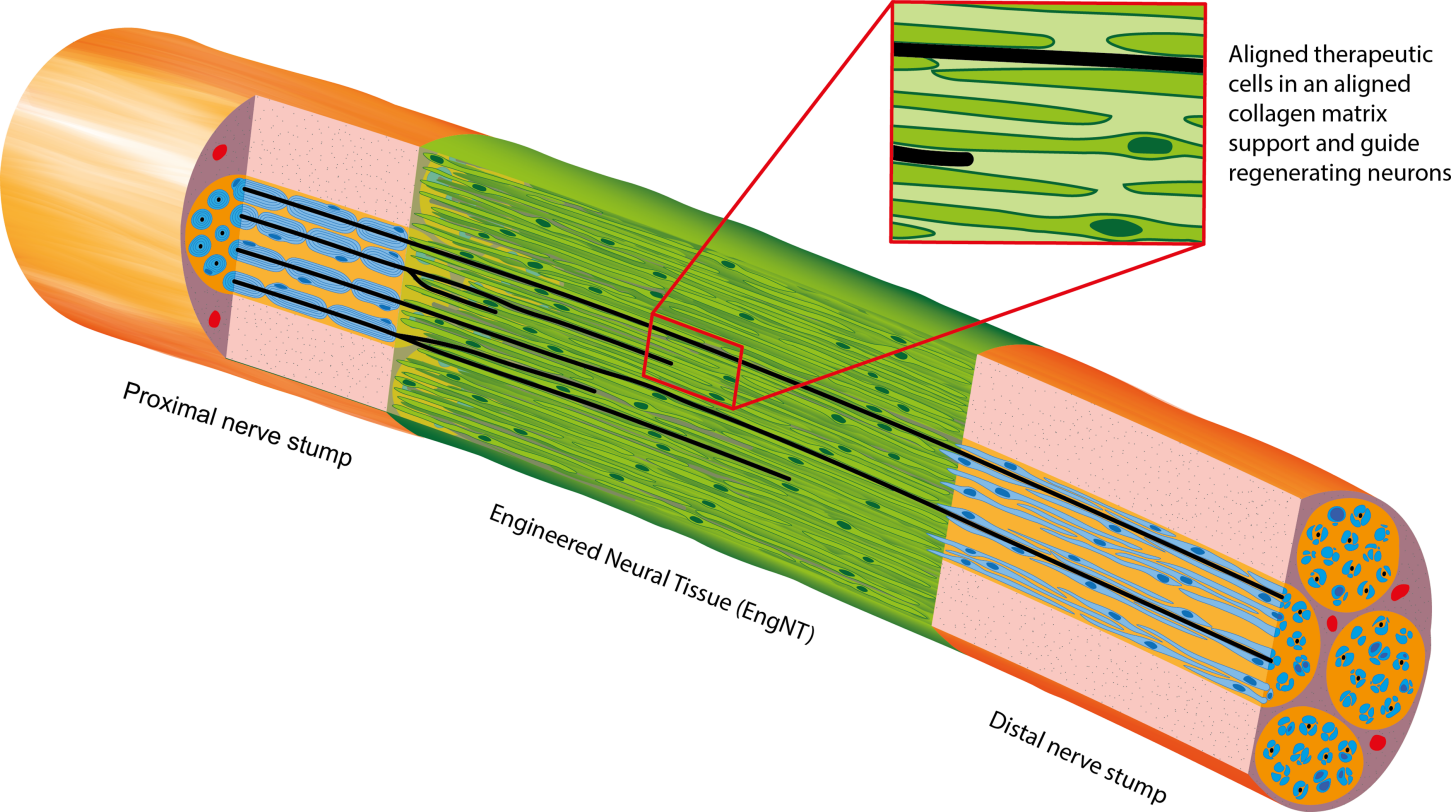
Engineered neural tissue can be implanted to support regeneration of neurons across a gap in a damaged peripheral nerve. The living EngNT material contains highly aligned elongated cells embedded within an aligned hydrogel matrix. Once implanted, it forms a bridge between the proximal and distal nerve stumps, supporting and guiding neuronal regeneration across the gap. Figure supplied by Sarah Hannis and reproduced with permission from [4].

Engineered Neural Tissue (EngNT) aims to recreate key features of the nerve graft, using columns of elongated therapeutic cells embedded within an aligned collagen matrix to form a living structure to support and guide regeneration (Figure 1). The tissue engineering technology has been described in detail along with extensive evidence demonstrating the ability of EngNT constructs to support and guide neuronal regeneration *in vitro* and *in vivo* [19–25]. The ongoing challenge therefore is not how to build engineered neural tissue, or whether it shows preclinical efficacy, but how to overcome hurdles associated with translation to clinical application.

## Current thinking and future directions

### Cellular component

Cell therapy has advanced considerably since EngNT was first conceived and consequently iterations of the technology have explored the feasibility of different cell sources. After initial development using rat Schwann cells [19], stem cells derived from adipose tissue [20], dental pulp [24] and bone marrow [21] were tested. More recently a clinical-grade human neural stem cell line was used, providing the opportunity for an off-the-shelf allogeneic approach [22]. Advances in methods for obtaining and controlling the differentiation of pluripotent cells provide new opportunities to improve the cellular component [26], especially since the greater understanding of repair phenotype Schwann cells and the role of endothelial cells is helping to inform the target cell phenotype [6,27–31]. Continued improvement in cell therapy technologies, including *ex vivo* gene therapy, provide opportunities to refine further the cellular component of EngNT [32,33]. The ideal cell would be well characterised and easy to manufacture, integrate without adverse host responses, support and guide regenerating neurites within the graft but not hinder their exit at the distal interface, survive hypoxia and ischaemia during integration and carry no risk of tumour formation, while being amenable to commercial-scale application.

### Materials and manufacture

At some point there may be a synthetic hydrogel that outperforms the current collagen option for EngNT, but that opportunity has not yet emerged despite synthetic polymers being widely researched for nerve repair [12,13,34]. The disadvantages of using an animal-derived protein biomaterial are outweighed somewhat in EngNT since collagen forms the bulk extracellular matrix of natural nerve tissue, therefore EngNT integrates without the challenges of degradation products and mechanical mismatch associated with other biomaterials. Materials and manufacturing approaches have been developed that allow EngNT to be produced in accordance with Good Manufacturing Practice, either using the traditional cellular self-alignment and stabilisation method [19] or a newer Gel Aspiration and Ejection approach [35]. Since EngNT resembles the endoneurium, it is delivered within a thin but strong outer sheath which mimics key mechanical features of the outer epineurium and perineurium, providing overall mechanical properties that match the host nerve tissue. Current manufacturing and assembly approaches are adequate for early stage clinical trials involving low numbers of constructs, with significant opportunities for new automated production technology to enable manufacture at scale [36]. Future developments are likely to involve optimising the density and distribution of cells within EngNT using mathematical modelling [37], and perhaps tuning local stiffness to enhance regeneration and integration [38].

### Commercialisation and clinical translation

For EngNT to move from biological proof of concept to clinical adoption there must be a commercial opportunity sufficient to attract the investment required for translation. Many hurdles have already been overcome — the use of cells and materials suitable for allogeneic ‘off-the-shelf’ application avoids the expense, variability and delay associated with using autologous cells [22]. Increased use of cell and gene therapies to treat a wide range of conditions has driven the expansion of the ATMP industry sector and associated manufacturing [39], reducing production costs to levels that will permit widespread adoption. The ability to cryopreserve EngNT for storage and transportation removes many logistical barriers [40]. Regulators are becoming more experienced with ATMPs which provides a clear route through preclinical and clinical testing stages [39]. In addition to the technological developments associated with EngNT and other ATMPs, the emergence of decellularized nerve allograft products alongside the various nerve repair conduits has galvanised interest in this sector, yielding useful research into market sizes and information about commercial opportunities.

There has also been renewed interest in the clinical evaluation of nerve repair products [32]. Measuring efficacy has traditionally been a problematic issue because nerve injuries are diverse in nature, rarely exist in isolation, and measurable outcomes are often slow and subjective. Recent advances have improved clinical trial approaches for peripheral nerve interventions, providing opportunities to evaluate EngNT and other new therapies in a robust objective manner over an acceptable time frame [12,41–44]. Consequently, opportunities exist for early clinical evaluation of EngNT to be conducted in patients with digital nerve injuries, or at the donor site in patients undergoing autografting.

## Conclusions

EngNT has scientific, commercial and clinical potential to be used in the repair of peripheral nerve injuries. While the obvious application is in bridging long gap injuries, by removing the limitations associated with autograft availability and morbidity it also opens up a wider range of opportunities. For example, having readily available living nerve tissue could improve outcomes in shorter gaps where autografting would currently be beneficial but not justifiable, or outside specialist referral centres where the expertise, infrastructure or time required for autograft is not available, or where unplanned iatrogenic nerve injury would benefit from immediate repair [45]. Other scenarios beyond peripheral nerve might include the treatment of optic nerve and spinal cord injury.

Current EngNT technology has been shown to match autograft performance in rat models [46] and the challenges of making a clinical-grade version for evaluation and future commercial manufacture have been addressed. Future modifications and improvements are likely to emerge from advances in cell engineering to enhance the phenotype of the cellular component, and optimisation of distribution patterns of cells and materials using mathematical modelling approaches. From an industry perspective, the idea of using an ATMP of this kind to repair nerves is novel and therefore carries uncertainty, but feasibility is constantly improving as advanced therapies become more widely adopted and the nerve repair market becomes better understood. For some time now we have had a sufficient understanding of the autograft to be able to recreate the key features artificially. EngNT uses a novel but pragmatic approach to create living replacement nerve tissue suitable for clinical-grade manufacture and the company Glialign Ltd (London, U.K.) has been established to take the technology forwards.

## Open Access

Open access for this article was enabled by the participation of University College London in an all-inclusive *Read & Publish* pilot with Portland Press and the Biochemical Society under a transformative agreement with JISC.

## Abbreviations

ATMPs: Advanced Therapy Medicinal Products
EngNT: Engineered Neural Tissue
